# Chronobiological variables predict non-response to serotonin and noradrenaline reuptake inhibitors in fibromyalgia: a cross-sectional study

**DOI:** 10.1007/s00296-024-05650-0

**Published:** 2024-07-29

**Authors:** Anna J. Krupa, Adrian A. Chrobak, Zbigniew Sołtys, Mariusz Korkosz, Jarosław Nowakowski, Dominika Dudek, Marcin Siwek

**Affiliations:** 1 Department of Affective Disorders, Jagiellonian University Medical College, Kopernika 21a, Krakow, 31- 501 Poland; 2 Department of Adult Psychiatry, Jagiellonian University Medical College, Krakow, Poland; 3 Laboratory of Experimental Neuropathology, Institute of Zoology and Biomedical Research, Jagiellonian University, Krakow, Poland; 4 Department of Rheumatology and Immunology, Jagiellonian University Medical College, Krakow, Poland

**Keywords:** Fibromyalgia, Chronotype, Circadian rhythm, Sleep-wake cycle, Sleep quality, Antidepressants

## Abstract

Available data shows associations between chronotype, circadian rhythms, sleep quality and fibromyalgia (FM) presentation. However, no studies have explored links between the chronobiological variables and effectiveness of pharmacotherapy. We aimed to assess the chronotypes, circadian rhythms, sleep-wake cycle and sleep quality in FM and their links to treatment response to serotonin and noradrenalin reuptake inhibitors (SNRI). 60 FM patients: 30 responsive to SNRI (FM T[+]), 30 non-responsive to SNRI (FM T[-]) and 30 healthy controls participated. Subjects were assessed by physician and with questionnaire tools: Composite Scale of Morningness, Biological Rhythms Interview of Assessment in Neuropsychiatry, Sleep-Wake Pattern Assessment Questionnaire, Pittsburgh Sleep Quality Index and Fibromyalgia Impact Questionnaire. ANOVA analysis and simple logistic regressions were used to examine the relationships between chronological variables and response to SNRI. FM T[-] vs. FM T[+] presented lower morning affect (11.50[95%CI 9.96-13.04] vs. 14.00[95%CI 12.42-15.57];p=0.04), anytime wakeability (2.27[95%CI 1.4-3.13] vs. 4.03[95%CI 2.99-5.08];p=0.013) worse overall (11.40[95%CI 9.92-12.88] vs. 7.97[95%CI 6.75-9.19];p=0.002) and subjective (1.70[95%CI 1.30-2.01] vs. 1.17[95%CI 0.94-1.39];p=0.008) sleep quality, higher circadian rhythm disruptions (55.47[95%CI 52.32-58.62] vs. 44.97[95%CI 41.31-48.62];p<0.001), sleep disturbances (1.63[95%CI 1.38-1.68] vs. 1.30[95%CI 1.1-1.5];p=0.04), sleeping-medication use (1.80[95%CI 1.27-2.32] vs. 0.70[95%CI 0.28-1.12];p=0.003). Levels of morningness (AIC=82.91,OR=0.93,p=0.05), morning affect (AIC=81.901,OR=0.86,p=0.03) diurnal dysrhythmia (AIC=69.566,OR=1.14,p<0.001), anytime wakeability (AIC=80.307,OR=0.76,p=0.015), overall sleep quality (AIC=74.665, OR=1.31,p=0.002) subjective sleep quality (AIC=79.353, OR=2.832,p=0.01) and disturbances (AIC=82.669,OR=2.54,p=0.043), sleep medication use (AIC=77.017, OR=1.9,p=0.003) and daytime disfunction (AIC=82.908, OR=1.971,p=0.049) were predictors of non-response to SNRI. Chronobiological variables vary between FM T[+] and FM T[-] and are predictors of non-response to SNRI.

**Supplementary Information:**

The online version contains supplementary material available at 10.1007/s00296-024-05650-0.

## Introduction

Fibromyalgia (FM) is a rather common disorder affecting 2–4% of the general population. The generalized pain is its central symptom, but sleep problems, fatigue, stiffness, depression, anxiety and cognitive impairment are also prevalent [1, 2]. The pathophysiology and treatment of FM remain under-researched, which obstructs the personalization of FM management and results in impaired functioning and quality of life of the affected individuals [3–5] and significant economic costs for society [6, 7]. Serotonin and noradrenaline reuptake inhibitors (SNRI) are among the most robustly studied pharmacotherapeutic management options for FM [8]. Previous works have reported on the differences observed between FM patients who are responsive to SNRI or non-responsive to SNRI therapy in the domains of: FM clinical presentation and severity of diurnal rhythm disruptions [9], insulin resistance, psychological variables [10], and psychiatric comorbidities [11–13].

Indeed, some studies corroborate the relationships between circadian rhythms and FM and its clinical presentation. Rizzi et al. reported that EEG activity in FM presented a higher cyclic alternating pattern (instability of the level of vigilance) than in healthy controls (HC) and that its rates correlated with the number of tender points and sleep inefficiency [14]. Actigraphy studies have revealed that FM patients present higher levels of nighttime activity than HC [15]. Neikrug et al. [16] reported that the patterns of activity levels (assessed with actigraphy) were linked to the levels of fatigue, sleep parameters, and functioning in FM subjects. Caumo et al. [17] noted differences in the circadian rhythm of urinary secretion of melatonin metabolite in participants with FM and major depressive disorder (MDD) vs. HC. They also observed that in FM, the pattern of melatonin secretion was correlated with pain threshold, number of trigger points, depressive symptoms, and sleep quality. Bulbul et al. [18] showed that diurnal rhythm disruption was correlated with higher severity of FM symptoms assessed Fibromyalgia Impact Questionnaire (FIQ) scores and severity of pain, depression as well as impaired sleep quality. In addition, Ucar et al. [19] described the correlations between impaired circadian rhythms and the severity of FM and depression. Few studies have explored the chronotypes in FM and their relationship with the disorder. Türkoğlu and Selvi [20] found, that compared to morning types, evening types showed higher FIQ scores, sleep problems, depression, and anxiety as well as lower quality of life. Likewise, Kantermann et al. [21] noted that, compared to FM patients with early chronotypes, those with late chronotypes reported higher levels of FM impact, perceived stress, and depression. Interestingly, a study in subjects with musculoskeletal pain revealed that the reduction of health-related quality of life was more pronounced in participants with evening vs. morning preference [22] and that evening types were more likely to suffer “disabling” musculoskeletal pain than early types but only after controlling the association for mental distress and insomnia [23]. The cited studies explored chronotype in FM mainly in the morningness-eveningness dimension. However, there is a more complex understanding of the sleep-wake cycle proposed by Putilov and Putilov [24], which assesses three parameters of chronotype (evening and morning lateness), plasticity (anytime and daytime wakeability), and vigor (anytime and nighttime sleepability), which have not yet been evaluated in the FM population. Moreover, aside from the above-mentioned preliminary report of our work, there are no data on the possible associations between chronotypes, circadian rhythms, sleep-wake cycle, and treatment response to SNRI in FM. The body of research indicating that, compared to HC, patients with FM, have impaired sleep quality is rather extensive [25–28]. Some studies have shown that sleep quality is linked to the severity of FM as assessed by the FIQ [20], pain intensity and extent [29], pain threshold [30], depression [31], anxiety, and FM impact [32]. More recently, it was also found that sleep disturbance mediates the effect of FM on cognitive performance [33] and that higher severity of insomnia in FM is associated with the use of centrally acting medications (e.g., anticonvulsants or SNRI) [34]. Still, little is known about the association between sleep quality and the effectiveness of SNRI in FM.

This work aimed to explore the chronotypes, circadian rhythms, sleep-wake cycle, and sleep quality in FM and their links to the treatment response to SNRI in FM.

## Methods

This paper is based on an observational, cross-sectional study.

### Patients

The inclusion and exclusion criteria, inclusion and exclusion criteria; criteria for assessment of the main outcome variable which was the response to SNRI treatment, definitions of response or non-response to treatment as well as participants group demographic data were previously described in [10].

### Main outcome variable

Response to SNRI treatment was the main outcome variable in this study. According to the earlier reported criteria [10] participants were assigned to two groups: either responsive (FM [T+]) or non-responsive (FM [T-]) to treatment with SNRI.

### Study factors

All subjects underwent an interview with a physician and completed the self-assessment scales. FM severity was assessed using the FIQ [35], Widespread Pain Index (WPI), Symptom Severity Scale (SSI), and Fibromyalgia Severity (FS) [1].

### All subjects filled out the self-report questionnaires to evaluate

1. chronotype the Composite Scale of Morningness (CSM). The CSM measures diurnal preference. The CSM is divided into two components: morning affect and circadian preference [36, 37].
2. circadian rhythms alterations: the Biological Rhythms Interview of Assessment in Neuropsychiatry (BRIAN). The BRIAN assesses biological rhythms in bipolar spectrum disorders but was also used in MDD and FM research [18, 19, 38]. The BRIAN presents external validity compared to objective parameters of rhythmicity [39–41].
3. sleep-wake patterns the Sleep-Wake Pattern Assessment Questionnaire (SWPAQ). The SWPAQ measures: evening and morning lateness, anytime and daytime wakeability, anytime and night-time sleepability [42, 43].
4. sleep quality the Pittsburgh Sleep Quality Index (PSQI). The PSQI assesses: subjective sleep quality, habitual sleep efficiency, sleep latency, sleep duration, sleep disturbances, use of sleep medication, and daytime dysfunction [44].

### Other variables

The data on other assessed variables were presented in [10, 11].

### Procedures

All participants signed an informed written consent. The study was approved by a local bioethical committee (No. 1072.6120.172.2021 issued on 25.11.2020) and performed in accordance with the principles of the Declaration of Helsinki. The authors obtained permission to use questionnaires if needed.

Given that some of the descriptive data from this study have already been published [10], we briefly reported the most relevant information in the main body of the text and enclosed the already available elsewhere demographic data and report on FM presentation as supplementary materials.

### Statistical analysis

All data were tested for normality and homogeneity of variances using the Shapiro-Wilk and Levene tests. Demographic data were compared between the groups using Student t-test for the quantitative variables or chi-squared for the qualitative variables. To compare the levels of morningness-eveningness, diurnal rhythms disruptions, features of sleep-wake cycle, and sleep quality in all studied groups and the severity of FM in the patient groups one-way analysis of variance (ANOVA) or Kruskal-Wallis tests were conducted. P-values lower than 0.05 were considered significant. Moreover, post-hoc tests (Tukey, Games-Howell, or pairwise Mann-Whitney with Benjamini-Hochberg correction) and effect size calculations (r, Hedges g, eta-squared) were performed. The associations between morningness-eveningness, circadian rhythm disturbances, sleep-wake cycle, sleep quality, and the lack of response to SNRI treatment were evaluated using a series of simple logistic regression analyses. Because of high correlations between chronobiological variables it was not possible to build a regression model with two or more independent variables. Statistical analyses were performed using the R software [45], mainly with functions from the rstatix and stats packages.

## Results

99 FM patients were invited to take part this study; 21 were not enrolled because did not meet the inclusion criteria; 18 were not enrolled due to lack of their consent to participate in the study.

A total of 90 participants were recruited in this study (30 FM T [+], 30 FM T [-], and 30 HC).

### Demographic data

The groups were comparable in terms of sex, age, and comorbidities. The mean BMI was higher in the whole FM group than in the HC and in FM T [-] than in the HC and FM T[+]. Mean BMI was comparable between HC and FM T [+]. The proportion of smokers was similar in HC vs. FM and HC vs. FM T [+]. The proportion of smokers was higher in FM T [-] vs. HC and FM T [+] (Supplementary materials Table S1).

### Fibromyalgia clinical presentation

FM presentation varied between FM T [+] and FM T [-]. FM T [-] showed longer duration of illness (*p* = 0.03), higher FIQ total score (*p* < 0.001), SSS (*p* < 0.001) and FS (*p* = 0.004), and higher scores of impairment of physical functioning (*p* < 0.001), work (*p* < 0.001) and well-being (*p* = 0.015) than FM T [+]. Pain (*p* = 0.02) levels were higher in FM T [-] vs. FM T [+]. The levels of stiffness showed a trend toward higher levels in FM T[-] vs. FM T[+] (*p* = 0.05). The FM subgroups were comparable regarding the length of time between onset of symptoms and diagnosis; score of FIQ fatigue/sleep and psychological symptomatology as well as WPI score (Supplementary materials Table S1).

### Chronobiological variables

#### Morningness-eveningness

Compared to HC, the FM group as a whole (*p* < 0.001) showed lower morningness measured with the CSM total score, morning affect (*p* < 0.001), and circadian preference (*p* < 0.003) subscales. Both FM T [+] and FM T [-] presented lower levels of overall morningness (*p* = 0.037 and *p* < 0.001), morning affect (*p* = 0.025 and *p* < 0.001), and morning circadian preference (*p* = 0.04 and *p* = 0.009). Moreover, significantly lower levels of morning affect were observed in FM T[-] vs. FM T [+] (*p* = 0.04, Table 2).

#### Circadian rhythm disruption

The level of diurnal dysrhythmia assessed with BRIAN was significantly higher in the whole FM group vs. HC (*p* < 0.001). Both FM T [+] (*p* < 0.001) and FM [-] (*p* < 0.001) showed higher aberrations of circadian rhythm than HC, furthermore the FM T [-] presented more pronounced disruption of diurnal rhythms than FM T [+] (*p* < 0.001)(Table 2). The level of evening chronotype as measured by BRIAN was higher in FM vs. HC (*p* = 0.042) and FM T[-] vs. HC (*p* = 0.008) (Table 2).

#### Sleep-wake cycle

According to the SWPAQ, no significant differences were noted between the groups regarding eveningness or anytime sleepability. The FM group as a whole (*p* < 0.001), as well as both FM T [+] (*p* = 0.018) and FM T [-] (*p* = 0.005) showed higher morning lateness compared to HC. There was no significant difference between FM T [+] and FM T [-] in morning lateness (*p* = 0.71). FM as the whole group (*p* < 0.001) and FM T [-] (*p* < 0.001) presented lower nighttime sleepability than HC. No significant difference in nighttime sleepability was noted between FM T [+] vs. HC (*p* = 0.051) or FM T [-] (*p* = 0.06). The FM as a whole (*p* < 0.003) and FM T [+] (*p* = 0.003) showed lower daytime wakeability than HC, while the differences between FM T [-] and HC (*p* = 0.08) or FM T [+] and FM T [-] (*p* = 0.17) were not significant. The FM as a whole (*p* < 0.003) and FM T [-] (*p* < 0.001) presented lower anytime wakeability compared to HC. FM T [-] vs. FM T [+] had lower anytime wakeability (*p* = 0.013), whereas the differences between FM T [+] and HC (*p* = 0.12) were not significant (Table 1).

**Table 1:** Comparisons of chronobiological variables between studied groups

Variable	HC mean score and 95% CI	FM mean score and 95% CI	T or Mann-Whitney test, HC vs. FM, effect size*,**	FM T [+] mean score and 95% CI	FM T [-] mean score and 95% CI	ANOVA or Kruskal-Wallis test, effect size**	HC vs. FM T [+]	HC vs. FM T [-]	FM T [+] vs. FM T [-]
CSM sum	39.20 [36.83, 41.57]	32.50 [30.48, 34.52]	t(88) = 4.064 *p* < 0.001 g = 0.90	34.53 [31.78, 37.28]	30.47 [27.43, 33.4]	F(2, 87) = 10.987 *p* < 0.001 η^**2**^ = 0.2	*p* = 0.037	*p* < 0.001	*p* = 0.08
CSM morning affect	16.70 [15.44, 17.96]	12.75 [11.63, 13.87]	t(88) = 4.371 *p* < 0.001 g = 0.97	14.00 [12.42, 15.57]	11.50 [9.96, 13.04]	F(2, 87) = 13.167 *p* < 0.001 η^**2**^ = 0.23	*p* = 0.025	*p* < 0.001	*p* = 0.04
CSM circadian preference	22.50 [21.18, 23.81]	19.75 [18.57, 20.93]	W = 1251.5 *p* < 0.003 *r* = 0.32	20.53 [19.15, 21.91]	18.97 [17.01, 20.93]	χ^2^ (2) = 10.314 *p* < 0.006 η^**2**^ = 0.1	*p* = 0.04	*p* = 0.009	*p* = 0.21
BRIAN sum	36.43 [33.66, 39.21]	50.22 [47.51, 52.93]	t(88) = -6.426 *p* < 0.001 g = 1.64	44.97 [41.31, 48.62]	55.47 [52.32, 58.62]	F(2, 87) = 36.814 *p* < 0.001 η^**2**^ = 0.46	*p* < 0.001	*p* < 0.001	*p* < 0.001
BRIAN chronotype	4.87 [4.2, 5.54]	6.08 [5.41, 6.75]	W = 665.5 *p* < 0.042 *r* = 0.22	5.50 [4.52, 6.48]	6.67 [5.75, 7.58]	χ^2^ (2) = 8.45 *p* < 0.015 η^**2**^ = 0.07	*p* = 0.63	*p* = 0.008	*p* = 0.1
SWPAQ evening leateness	3.63 [2.47, 4.79]	4.90 [3.86, 5.94]	W = 764 *p* = 0.24 *r* = 0.12	4.07 [2.86, 5.27]	5.73 [4.02, 7.44]	χ^2^ (2) = 2.37 *p* = 0.31 η^**2**^ = 0.004	*p* = 0.56	*p* = 0.44	*p* = 0.44
SWPAQ antytime sleepability	5.10 [3.91, 6.29]	5.93 [5.1, 6.77]	W = 770 *p* = 0.26 *r* = 0.12	6.70 [5.6, 7.8]	5.17 [3.91, 6.42]	χ^2^ (2) = 5.023 *p* = 0.08 η^**2**^ = 0.04	*p* = 0.09	*p* = 0.97	*p* = 0.09
SWPAQ morning lateness	5.27 [4.01, 6.52]	7.80 [7.01, 8.59]	W = 518 *p* = 0.001 *r* = 0.35	7.57 [6.32, 8.81]	8.03 [6.99, 9.08]	χ^2^ (2) = 11.002 *p* = 0.004 η^**2**^ = 0.1	*p* = 0.018	*p* = 0.005	*p* = 0.71
SWPAQ nighttime sleepability	7.73 [6.43, 9.03]	4.87 [3.97, 5.76]	W = 1289.5 *p* < 0.001 *r* = 0.35	5.70 [4.38, 7.02]	4.03 [2.82, 5.24]	χ^2^ (2) = 14.385 *p* < 0.001 η^**2**^ = 0.14	*p* = 0.051	*p* < 0.001	*p* = 0.06
SWPAQ daytime wakeability	5.97 [4.99, 6.94]	4.15 [3.47, 4.83]	W = 1250 *p* < 0.003 *r* = 0.32	3.70 [2.78, 4.62]	4.60 [3.57, 5.63]	χ^2^ (2) = 10.949 *p* = 0.004 η^**2**^ = 0.1	*p* = 0.003	*p* = 0.08	*p* = 0.17
SWPAQ anytime wakeability	5.50 [4.13, 6.87]	3.15 [2.45, 3.85]	W = 1245 *p* < 0.003 *r* = 0.31	4.03 [2.99, 5.08]	2.27 [1.4, 3.13]	χ^2^ (2) = 14.801 *p* < 0.001 η^**2**^ = 0.15	*p* = 0.12	*p* < 0.001	p = **0.013**
PSQI sum	4.03 [3.18, 4.89]	9.68 [8.65, 10.71]	W = 181.5 *p* < 0.001 *r* = 0.65	7.97 [6.75, 9.19]	11.40 [9.92, 12.88]	χ^2^ (2) = 44.969 *p* < 0.001 η^**2**^ = 0.49	*p* < 0.001	*p* < 0.001	*p* = 0.002
PSQI subjective sleep quality	0.73 [0.52, 0.92]	1.43 [1.23, 1.63]	W = 465 *p* < 0.001 *r* = 0.43	1.17 [0.94, 1.39]	1.70 [1.39, 2.01]	χ^2^ (2) = 22.518 *p* < 0.001 η^**2**^ = 0.24	*p* = 0.008	*p* < 0.001	*p* = 0.008
PSQI sleep latency	0.70 [0.43, 0.92]	1.82 [1.57, 2.07]	W = 351.5 *p* < 0.001 *r* = 0.51	1.60 [1.25, 1.95]	2.03 [1.66, 2.41]	χ^2^ (2) = 26.316 *p* < 0.001 η^**2**^ = 0.28	*p* < 0.001	*p* < 0.001	*p* = 0.07
PSQI sleep duration	0.50 [0.16, 0.84]	0.82 [0.54, 1.09]	W = 760.5 p **=** 0.17 *r* = 0.14	0.57 [0.16, 0.84]	1.07 [0.54, 1.09]	χ^2^ (2) = 6.11 *p* = 0.048 η^**2**^ = 0.05	*p* = 0.91	*p* = 0.08	*p* = 0.08
PSQI habitual sleep	0.20 [0.05, 0.35]	0.95 [0.66, 1.24]	W = 564 *p* = 0.001 *r* = 0.34	0.90 [0.52, 1.28]	1.00 [0.55, 1.45]	χ^2^ (2) = 10.561 *p* = 0.005 η^**2**^ = 0.1	*p* = 0.007	*p* = 0.007	*p* = 0.91
PSQI sleep disturbances	0.93 [0.8, 1.07]	1.47 [1.3, 1.63]	W = 490 *p* < 0.001 *r* = 0.43	1.30 [1.1, 1.5]	1.63 [1.38, 1.88]	χ^2^ (2) = 2.171 *p* < 0.001 η^**2**^ = 0.22	*p* = 0.005	*p* < 0.001	*p* = 0.04
PSQI sleeping medication	0.13 [-0.08, 0.35]	1.25 [0.9, 1.6]	W = 502.5 *p* < 0.001 *r* = 0.42	0.70 [0.28, 1.12]	1.80 [1.27, 2.32]	χ^2^ (2) = 25.866 *p* < 0.001 η^**2**^ = 0.27	*p* = 0.011	*p* < 0.001	*p* = 0.003
PSQI daytime disfunction	0.83 [0.59, 1.08]	1.95 [1.73, 2.16]	W = 284 *p* < 0.001 *r* = 0.58	1.73 [1.39, 2.07]	2.17 [1.91, 2.43]	χ^2^ (2) = 33.769 *p* < 0.001 η^**2**^ = 0.36	*p* < 0.001	*p* < 0.001	*p* = 0.07

BRIAN- Biological Rhythms Interview for Assessment in Neuropsychiatry; CI - Confidence Interval; CSM- Composite Scale of Morningness; FM- fibromyalgia patients as a whole group; FM T [+]- patients responsive to SNRI treatment; FM T [-]- patients resistant to SNRI treatment; HC- healthy controls; PSQI- Pittsburgh Sleep Quality Index; SWPAQ- Sleep-Wake Pattern Assessment Questionnaire

*g- Hedges g is the measure of effect size for t test. Effect size lower than 0.2 was counted as negligible. 0.2–0.5 as small. 0.5–0.8 as moderate and for 0.8 as large

** r is the measure of effect size for Mann-Whitney test. Interpretation: < 0.3 - small, 0.3–0.5 moderate, > 0.5 large effect

***η^2^ - (eta squared) is the measure of effect size for more than two groups comparison. Effect size lower than 0.01 was counted as negligible. 0.01–0.06 as small. 0.06–0.14 as moderate and higher than 0.14 as large

#### Sleep quality

The quality of sleep assessed as the total score of the PSQI (*p* < 0.001) as well as the sleep quality subscale (*p* < 0.001) were significantly lower in FM vs. HC, as well as FM T [+] vs. HC (*p* = 0.008 and *p* < 0.001), and FM T [-] vs. HC (*p* < 0.001 and *p* < 0.001). In addition, the overall and subjective quality of sleep was significantly lower in FM T [-] than in FM T [+] (*p* = 0.002 and *p* = 0.008). The scores of sleep latency, habitual sleep, sleep disturbances, and sleep medication were all higher in FM vs. HC (*p* < 0.001; *p* = 0.001; *p* < 0.001; *p* < 0.001); FM T [+] vs. HC (*p* < 0.001, *p* = 0.007, *p* = 0.005, *p* = 0.0011, *p* < 0.001); FM T [-] vs. HC (*p* < 0.001, *p* = 0.007, *p* < 0.001, *p* < 0.001, *p* < 0.001). FM T [-] presented higher levels of sleep disturbance (*p* = 0.04) and sleeping medication use (*p* = 0.003) than FM T [+]. The severity of daytime dysfunction was higher in FM vs. HC (*p* < 0.001), FM T [+] vs. HC (*p* < 0.001), and FM T [-] vs. HC (*p* < 0.001). The comparison of daytime dysfunction between FM T [+] and FM T [-] did not reach statistical significance (*p* = 0.07). No significant differences were noted among the studied groups concerning the duration of sleep (Table 1).

### Associations between chronobiological variables and lack of response to SNRI treatment

Logistic regression analysis indicated that (1) lower morningness measured by CSM total score (*p* = 0.05) and morning affect subscale (*p* = 0.03), (2) higher diurnal rhythm disruption as assessed by BRIAN (*p* < 0.001), (3) lower anytime wakeability as indicated by SWPAQ (*p* = 0.015), and (4) lower overall sleep quality as measured by PSQI sum (*p* = 0.003) and the subscales of subjective sleep quality (*p* = 0.01), sleep disturbances (*p* = 0.043), sleeping medication (*p* = 0.003) and daytime dysfunction (*p* = 0.049) were significant predictors of lack of response to SNRI treatment in FM (Table 2).

**Table 2:** Simple logistic regressions evaluating the associations between chronobiological variables and lack of response to SNRI treatment

Variable	Intercept	Slope	*p*	AIC	OR	2.50%	97.50%	*p*
CSM sum	2.354	-0.073	**0.05**	82.91	0.93	0.86	0.996	**0.05**
CSM morning affect	1.855	-0.146	**0.03**	81.901	0.864	0.750	0.979	**0.03**
CSM circadian preference	1.554	-0.079	0.19	85.357	0.924	0.817	1.035	0.19
BRIAN sum	-6.614	0.131	**0.0006**	69.566	1.14	1.065	1.239	**< 0.001**
BRIAN chronotype	-1.108	0.183	0.08	84.035	1.2	0.981	1.492	0.08
SWPAQ evening lateness	-0.526	0.108	0.11	84.515	1.114	0.980	1.280	0.11
SWPAQ anytime sleepability	0.922	-0.156	0.07	83.665	0.856	0.718	1.007	0.07
SWPAQ morning lateness	-0.397	0.051	0.55	86.823	1.052	0.890	1.250	0.55
SWPAQ nighttime sleepability	0.708	-0.146	0.07	83.586	0.864	0.734	1.005	0.07
SWPAQ daytime wakeability	-0.561	0.136	0.19	85.368	1.145	0.941	1.414	0.19
SWPAQ anytime wakeability	0.839	-0.273	**0.015**	80.307	0.761	0.598	0.963	**0.015**
PSQI sum	-2.605	0.272	**0.003**	74.665	1.313	1.119	1.597	**0.002**
PSQI subjective sleep quality	-1.484	1.041	**0.01**	79.353	2.832	1.345	6.744	**0.01**
PSQI sleep latency	-0.86	0.472	0.09	84.161	1.603	0.942	2.844	0.09
PSQI sleep duration	-0.374	0.469	0.07	83.743	1.598	0.974	2.753	0.07
PSQI habitual sleep	-0.078	0.082	0.75	87.054	1.086	0.684	1.737	0.73
PSQI sleep disturbances	-1.366	0.932	**0.043**	82.669	2.54	1.072	6.607	**0.043**
PSQI sleeping medications	-0.782	0.641	**0.003**	77.017	1.9	1.271	2.956	**0.003**
PSQI daytime disfunction	-1.328	0.678	**0.049**	82.908	1.971	1.034	4.060	**0.049**

AIC - Akaike information criterion; BRIAN- Biological Rhythms Interview for Assessment in Neuropsychiatry; CSM- Composite Scale of Morningness; PSQI- Pittsburgh Sleep Quality Index; SWPAQ- Sleep-Wake Pattern Assessment Questionnaire; OR- odds ratio

## Discussion

Several studies have previously assessed the chronotype, sleep and circadian rhythm disturbances in FM and their associations with the severity of FM symptoms. The novelty of our work lies in the exploration of the chronobiological variables in relation to the lack of response to SNRI treatment. Based on the previously reported clinical, chronobiological, metabolic, and psychological heterogeneity of the FM group [9, 12, 13], we distinguished two subgroups of patients responsive or non-responsive to SNRI treatment. The reported results uphold our hypothesis that FM T [+] and FM T [-] vary in clinical presentation in that FM T [-] present (1) lower morning affect, (2) more pronounced diurnal rhythm disruption, (3) lower anytime wakeability, (4) lower sleep quality measured with total score and the subscale of PSQI, and (5) higher sleep disturbance and sleeping medication intake compared to FM T [+] (Table 1). Similarly to Neikrug et al., we noted that the level of eveningness was higher in FM vs. HC [16], also our results indicated that both FM T [+] and FM T [-] presented higher eveningness than HC but there was no significant difference in the overall eveningness between FM T [+] and FM T [-]. Türkoğlu and Selvi [20] and Kantermann et al. [21] found links between the evening chronotype and FIQ scores as well as psychological symptoms. Our results indicate that lower morningness is a predictor of a lack of response to SNRI treatment in FM. Knowledge on the relationship between chronotype and response to treatment with antidepressant medication is very limited and inconsistent. McGlashan et al. reported on the reduced efficacy of selective serotonin reuptake inhibitors (SSRI) in MDD patients with evening chronotype [46] and in an analysis of a small group of MDD patients, it was noted that eveningness and circadian rhythm disruption were linked to resistance to SNRI [47]. On the other hand, Xavier et al., who assessed a non-homogenous group of patients with MDD diagnosis taking various antidepressants (SSRI, SNRI, and others), did not observe significant associations between chronotype variables and treatment response to antidepressants [48]. No studies have assessed the relationship between chronotype and effectiveness of antidepressants in FM. Notably, Burgess et al. showed that morning light therapy, which advances circadian timing, effectively reduced the severity of FM [49]. Further studies are needed to unravel the relationships between chronotype and response to specific antidepressant medications, as well as the potential use of interventions advancing circadian rhythm. What is more, in accordance with Bulbul et al. [18] and Neikrug [16] we noted higher levels of diurnal rhythm aberrations in FM vs. HC. Additionally, our results showed that the level of circadian dysrhythmia is higher in FM T [+] vs. HC, FM T [-] vs. HC, and FM T [-] vs. FM T [+] (Table 2). Moreover, the severity of diurnal disturbance is a predictor of lack of response to SNRI. Interestingly, while several differences in sleep-wake variables were found between HC and FM, FM T [+], FM T [-] the comparison between FM T [+] and FM T [-] did not reach statistical significance. Nonetheless, the regression analysis indicated that lower anytime wakeability was a predictor of lack of response to SNRI. Much like previous authors [25–28], we found that FM showed significantly lower sleep quality measured with PSQI total score and all of the subscales (aside from sleep duration) compared to HC. What is more, these same differences in sleep quality were also noted in the comparisons between HC and both FM T [+] and FM T [-]. Of the FM T [+] vs. FM T [-] comparisons only the one of sleep medication use was significantly different. Still, the regression analysis indicated that lower overall sleep quality and more pronounced sleep disturbances, daytime disfunction, and higher sleeping medication use were all significant predictors of lack of response to SNRI. While previous works have shown that poor sleep quality in FM is linked to the severity of FM symptoms and FM duration, ours has also found an association with non-response to SNRI [50, 51]. In sum, our work shows that FM T [+] and FM T [-] present dissimilar chronobiological characteristics regarding chronotype, circadian rhythm disruption, and sleep quality. Moreover, higher eveningness, severity of diurnal dysrhythmia, lower anytime wakeability, and worse sleep quality are predictors of lack of response to SNRI in FM.

There are several limitations of our work: the relatively small number of participants, the fact that assessment of chronobiological variables based largely on subjective measures, the cross-sectional design of the study. Nevertheless, this study unraveled new data that can be of both scientific and clinical importance.

In conclusion, the results indicate that there are several significant differences in chronobiological variables between FM T [+] and FM T [-]. What is more, our work showed that chronobiological variables, amongst them some modifiable such as circadian rhythm disruptions or sleep quality, are predictors of lack of response to SNRI in FM. Consequently, we believe psychoeducation and other interventions such as exercise [52] for the improvement of sleep should be provided as a standard part of FM treatment and follow-up. Perhaps FM patients resistant to SNRI could benefit from morning light therapy [49]. Further studies are needed to explore interventions centered on advancement of circadian timing, normalization of circadian rhythms and improvement of sleep quality as potentiation strategy in patients resistant to SNRI treatment.

## Electronic supplementary material

Below is the link to the electronic supplementary material.

Supplementary Material 1

## Data Availability

All data relevant to the study are presented in the article or available as supplementary material. Data analyzed in the current study are available from the corresponding author upon reasonable request.

## Declarations

### Disclaimer

No part of this manuscript has been copied or published elsewhere in whole or in part in any language.
