# Supplementary material for: Chronobiological variables predict non-response to serotonin and noradrenaline reuptake inhibitors in fibromyalgia: a cross-sectional study
Source: Rheumatol Int. 2024 Jul 29;44(10):1987–95. doi: 10.1007/s00296-024-05650-0 (PMC11393292; doi:10.1007/s00296-024-05650-0)
Supplement: Supplementary file 1 — Supplementary Material 1 [file 296_2024_5650_MOESM1_ESM.docx]

Supplementary materials

Article title: Chronobiological predictors of response to pharmacotherapy in fibromyalgia

Journal name: Rheumatology International

Author names: Anna Julia Krupa, Adrian Andrzej Chrobak, Zbigniew Sołtys, Mariusz Korkosz, Jarosław Nowakowski, Dominika Dudek, Marcin Siwek

Corresponding author: Marcin Siwek M.D. Ph.D. Assoc. Prof.,

Department of Affective Disorders, Jagiellonian University Medical College,

Postal address: Kopernika 21a, 31-501 Krakow, Poland,

Email address: marcin.siwek@uj.edu.pl

Table S1. Demographic data

| Variable | HC  n=30 | FM  n=60 | FM T [+]   n=30 | FM T  [-]   n=30 | HC vs. FM * | All groups ** | HC  vs.  FM T[+] | HC  vs.  FM T[-] | FM T [+] vs.  FM T [-] |
| --- | --- | --- | --- | --- | --- | --- | --- | --- | --- |
| Age mean and CI, in years | 44.03 [39.27, 48.79] | 45.12 [42.33, 47.9] | 43.53  [39.5, 47.57] | 46.7 [42.71, 50.69] | t(88) = -0.423  p= 0.67 | F(2, 87) = 0.663 p= 0.52 | p= 0.98 | p= 0.64 | p= 0.53 |
| Height mean and CI, in cm | 166.33 [164.03, 168.64] | 167.2 [165.1, 169.3] | 166.9 [164.04, 169.76] | 167.5 [164.24, 170.74] | t(88) = -0.515  p= 0.6 | F(2, 87) = 0.178 p= 0.84 | p= 0.96 | p= 0.82 | p= 0.95 |
| Weight mean and CI, in kg | 67.45 [62.47, 72.43] | 76.46 [71.8, 81.13] | 72.03 [65.42, 78.65] | 80.88 [74.31, 87.46] | t(88) = -2.42  **p= 0.017** | F(2, 87) = 5.233  **p= 0.007** | p= 0.53 | **p=0.006** | p= 0.1 |
| BMI kg/m2, mean and CI | 24.23 [22.84, 25.62] | 27.23 [25.78, 28.68] | 25.78  [23.59, 27.97] | 28.68 [26.8, 30.56] | t(81) = -3.02  **p= 0.003** | ꭓ^2^ (2) = 12.12  **p= 0.003** | p= 0.43 | **p= 0.001** | **p= 0.04** |
| Sex female/ male | 26 / 4 | 51 / 9 | 25 / 5 | 26 / 4 | ꭓ^2^ (90, 1) < 0.001  p> 0.99 | ꭓ^2^ (90, 2) < 0.001  p= 0.91 | p> 0.99 | p> 0.99 | p> 0.99 |
| Hyper-lipidemia (yes) | 4 | 2 | 1 | 1 | ꭓ^2^ (90, 1 ) < 0.001  p = 0.2 | ꭓ^2^ (90, 2) = 3.21  p= 0.2 | p= 0.35 | p> 0.99 | p> 0.99 |
| Hyper-tension (yes) | 5 | 9 | 2 | 7 | ꭓ^2^ (90, 1) = 1.81  p= 0.18 | ꭓ^2^ (90, 2) = 3.42  p= 0.18 | p= 0.67 | p= 0.51 | p= 0.15 |
| Hypo-thyroidism (yes) | 3 | 11 | 8 | 3 | ꭓ^2^ (90, 1)< 0.001  p= 0.47 | ꭓ^2^ (90, 2) = 4.23  p= 0.18 | p= 0.18 | p> 0.99 | p= 0.18 |
| Asthma (yes) | 1 | 5 | 2 | 3 | ꭓ2 (90, 1) = 0.2  p= 0.65 | ꭓ2 (90, 2) = 1.1  p= 0.58 | p> 0.99 | p= 0.61 | p> 0.99 |
| Allergies (yes) | 1 | 5 | 3 | 2 | ꭓ2 (90, 1) = 0.2  p= 0.65 | ꭓ2 (90, 2) = 1.1  p= 0.58 | p= 0.61 | p >0.99 | p> 0.99 |
| Derma-toses (yes) | 0 | 5 | 1 | 4 | ꭓ2 (90, 1) = 1.3  p= 0.25 | ꭓ2 (90, 1) = 5.51  p= 0.06 | p> 0.99 | p= 0.12 | p= 0.35 |
| Smoking (yes) | 3 | 14 | 2 | 12 | ꭓ^2^ (90, 1) < 0.001  p= 0.21 | ꭓ^2^ (90, 2) = 13.2  **p= 0.002** | p> 0.99 | **p= 0.02** | **p= 0.006** |

FM- fibromyalgia patients as a whole group, FM T [+]- patients responsive to SNRI treatment, FM T [-]- patients resistant to SNRI treatment, HC- healthy controls, CI - 95% confidence intervals

*χ*^2^ test was used to compare the qualitative data. *T-test was used to assess the differences in quantitative data, **- ANOVA was used to assess the differences in quantitative data. In the case of BMI, due to deviation of normality in one group, nonparametric tests (Kruskal-Wallis, Mann-Whitney) tests were used, with Benjamini-Hochberg correction for multiple comparisons.

Table S2. Comparisons of FM characteristics between FM T [+] and FM T [-]

| Variable | FM | FM T [+] | FM T[-] | T or Mann-Whitney Tests | FM T [+] vs.  FM T [-] |
| --- | --- | --- | --- | --- | --- |
| Duration of illness, mean and CI, years | 12.62 [9.85, 15.38] | 9.1  [6.39, 11.81] | 16.13  [11.49, 20.77] | W = 302 | **p= 0.03** |
| Time form onset to diagnosis, mean and CI, years | 7.29  [5.44, 9.14] | 6.08  [3.51, 8.65] | 8.5  [5.77, 11.23] | W = 344 | p= 0.12 |
| FIQ sum, mean and CI | 50.5  [45.29, 55.71] | 40.18  [32.86, 47.49] | 60.82  [55.23, 66.4] | W = 171 | **p< 0.001** |
| FIQ physical functioning, mean and CI | 2.84  [2.21, 3.47] | 1.76  [1.05, 2.47] | 3.93  [3.02, 4.84] | W = 207 | **p< 0.001** |
| FIQ wellbeing, mean and CI | 6.16  [5.38, 6.92] | 5.18  [3.99, 6.37] | 7.15  [6.18, 8.12] | W = 287 | **p= 0.015** |
| FIQ work related, mean and CI | 9.01  [7.69, 10.32] | 6.47  [4.9, 8.04] | 11.54  [9.79, 13.29] | t(58) = -4.41 | **p< 0.001** |
| FIQ pain, mean and CI | 5.58  [5.04, 6.12] | 4.87  [4.14, 5.59] | 6.3  [5.54, 7.05] | W = 280 | **p= 0.02** |
| FIQ fatigue/ sleep, mean and CI | 12.72  [11.23, 14.21] | 11.27  [8.99, 13.54] | 14.17  [12.25, 16.08] | W = 328 | p= 0.07 |
| FIQ stiffness, mean and CI | 5.67  [4.84, 6.49] | 4.77  [3.46, 6.08] | 6.57  [5.59, 7.55] | W = 318 | **p= 0.05** |
| FIQ psychological symptoms, mean and CI | 8.63  [7.24, 10.02] | 7.73  [5.91, 9.55] | 9.53  [7.38, 11.69] | t(58) = -1.31 | p= 0.45 |
| WPI, mean and CI | 14.07  [12.95, 15.18] | 13.2  [11.39, 15.01] | 14.93  [13.59, 16.28] | W = 364 | p= 0.21 |
| SSS, mean and CI | 8.08  [7.37, 8.8] | 6.83  [5.88, 7.78] | 9.33  [8.44, 10.23] | W = 198 | **p< 0.001** |
| FS, mean and CI | 22.13  [20.56, 23.7] | 20  [17.67, 22.33] | 24.27  [22.33, 26.2] | W = 253 | **p= 0.004** |

CI - Confidence Intervals, FIQ- Fibromyalgia Impact Questionnaire, FM- fibromyalgia patients as a whole group, FM T [+]- patients responsive to SNRI treatment, FM T [-]- patients resistant to SNRI treatment, FS- Fibromyalgia Severity, HC- healthy controls, SSS- Symptom Severity Scale, WPI- Widespread Pain Index
